# Spinal Cord Involvement in Adult Mitochondrial Diseases: A Cohort Study

**DOI:** 10.3390/life12010005

**Published:** 2021-12-21

**Authors:** Guido Primiano, Paolo Mariotti, Ida Turrini, Cristina Sancricca, Andrea Sabino, Alessandra Torraco, Rosalba Carrozzo, Serenella Servidei

**Affiliations:** 1 Fondazione Policlinico Universitario A. Gemelli IRCCS, 00168 Rome, Italy; paolo.mariotti@policlinicogemelli.it (P.M.); ida.turrini@gmail.com (I.T.); cristinasancricca@me.com (C.S.); serenella.servidei@unicatt.it (S.S.); 2 Dipartimento Universitario di Neuroscienze, Università Cattolica del Sacro Cuore, 00168 Rome, Italy; andrea.sabino@unicatt.it; 3 Unit of Muscular and Neurodegenerative Disorders, Laboratory of Molecular Medicine, Bambino Gesù Children’s Hospital, IRCCS, 00146 Rome, Italy; alessandra.torraco@opbg.net (A.T.); rosalba.carrozzo@opbg.net (R.C.)

**Keywords:** mitochondrial diseases, spinal cord, MRI, biomarkers, mtDNA

## Abstract

The central nervous system is metabolically very demanding and consequently vulnerable to defects of the mitochondrial respiratory chain. While the clinical manifestations and the corresponding radiological findings of the brain involvement in mitochondrial diseases (e.g., stroke-like episodes, signal changes of the basal ganglia, cerebral and cerebellar atrophy) are well known, at present there are few data on the spinal-cord abnormalities in these pathologies, in particular in adult subjects. In this study, we present a cross-sectional cohort study on the prevalence and characterization of spinal-cord involvement in adult patients with genetically defined mitochondrial diseases.

## 1. Introduction

Mitochondrial diseases (MDs) are a common group of genetic disorders characterized by a primary defect in mitochondrial oxidative phosphorylation, which is the main source of cellular ATP, and are under the dual genetic control of mitochondrial DNA (mtDNA) and nuclear DNA (nDNA) working in concert [1]. Tissues with a high dependence on the oxidative metabolism are the most frequently affected. In particular, the central nervous system (CNS), including the brain and spinal cord, is metabolically very demanding and consequently vulnerable to defects of the mitochondrial respiratory chain [2]. While the clinical manifestations and the corresponding radiological findings of the brain involvement of mitochondrial diseases (e.g., stroke-like episodes, signal changes of the basal ganglia, cerebral and cerebellar atrophy) are well known [3,4], at present there are few data on the spinal-cord abnormalities in these pathologies, especially in adult subjects [5,6]. A detailed evaluation of this clinical aspect has only been performed for pediatric or juvenile patients in small case studies with a single phenotype or in retrospective studies [7,8,9].

Here, we present a cross-sectional cohort study on the prevalence and characterization of spinal-cord involvement in adult patients with genetically defined MDs.

## 2. Materials and Methods

We included all of the consecutive adult mitochondrial patients (18 years and above) who came to our hospital, and performed a brain and spinal-cord MRI. In particular, the protocol for the spinal cord included sagittal and axial T1 and T2 weighted images with contrast administration. Two neurologists with experience in neuroradiology independently reviewed the MRI images. All of the patients, evaluated by a neurologist specialized in mitochondrial medicine, had a defined mitochondrial disease based on molecular, morphological and biochemical analysis. The study was approved by the Ethics Committee of the Università Cattolica del Sacro Cuore (Rome, Italy; ID: 3754) and all of the participants signed an informed consent prior to inclusion. All procedures were conducted in compliance to the ethical standards laid down in the 1964 Declaration of Helsinki and its later amendments.

## 3. Results

### 3.1. Patients

Fifty one patients (mean age: 45, range 18–78; 33 females) were enrolled during this period (Table 1): 20 patients had progressive external ophthalmoplegia (PEO) associated with single mtDNA deletion (n = 8), recessive or dominant mutations in the nuclear genes *POLG* (n = 6), *TWNK* (n = 2) and *SLC25A4* (n = 1) or multiple mtDNA deletions without an identified nuclear gene defect (n = 3); 3 had *POLG* mutations and ataxia neuropathy spectrum (ANS); 11 patients carried the m.3243A > G mutation affected by mitochondrial encephalomyopathy, lactic acidosis, and stroke-like episodes (MELAS; n = 3), maternally inherited diabetes and deafness (MIDD; n = 6), cardiomyopathy (n = 1) and PEO (n = 1); 5 patients had the m.8344A > G mutation and myoclonic epilepsy with ragged-red fibers (MERRF); 3 patients had Leber hereditary optic neuropathy (LHON) (2 associated with m.14484T > C mutation e and 1 with m.11778G > A) and 1 was affected by Leigh syndrome (LS) with m.13513G > A mutation; 2 patients presented with autosomal dominant optic atrophy (ADOA) and mutations in the *OPA1* gene; 1 subject was diagnosed with leukoencephalopathy with brainstem and spinal cord involvement and lactate elevation (LBSL) and mutations in *DARS2* gene; 2 mitochondrial neurogastrointestinal encephalomyopathy (MNGIE) harbored recessive *TYMP* mutations; 3 patients had other mtDNA point mutations including m.8356T > G, m.8356T > C (both associated with the MERRF phenotype) and m.13042G > A.

### 3.2. Spinal-Cord Imaging Findings

**Figure 1:**
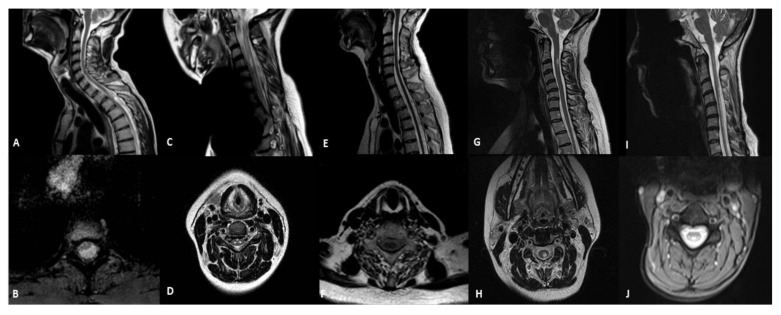
**Sagittal and axial T2-weithing images of MRI in patients with mitochondrial diseases and spinal cord involvement.** (**A,B**) MIDD with m.3243A > G mutation (patient 1): selective thinning of the spinal cord with a cystic-like lesion located in the white matter of dorsal and lateral columns; (**C**,**D**) Leigh syndrome with m.13513G > A (patient 2): hyperintensity spinal grey matter in the cervical and the upper-thoracic spinal cord segments and medullary atrophy; (**E**,**F**) ANS with *POLG* mutation (patient 3): medullary atrophy without signal alterations; (**G**,**H**) LHON with m.11778G > A (patient 4): focal hyperintensity in the cervical posterior columns; (**I**,**J**) LBSL with *DARS2* mutation (patient 5): abnormalities of the T2 signal of the cervical and thoracic spinal cord involving dorsal columns and lateral corticospinal tracts.

We found spinal cord abnormalities in five patients (9.8%; Table 2 and Figure 1). A 45-year-old man with the m.3243A > G mutation in the *MT-TL1* gene and the MIDD phenotype presented a significant thinning of the spinal cord at the level of the soma D3, where a focal area of signal hyperintensity was present in the T2 images with a cystic-like appearance located in the white matter of the dorsal and lateral columns (patient 1). The MRI showed symmetrical T2 hyperintensities in the spinal grey matter in the cervical and the upper-thoracic spinal-cord segments and a medullary atrophy in an 18-year-old girl with Leigh syndrome and m.13513G > A mutation (patient 2). A medullary atrophy without signal alteration was detected in one patient who was affected by ANS associated with a mutation in the *POLG* gene (c.3293A > G) (patient 3). The neuroimaging study in a 27-year-old female with the m.11778G > A mutation affected by LHON and clinically definite multiple sclerosis (MS) showed focal abnormalities in the dorsal and lateral columns of the spinal cord (patient 4). Finally, a 41-year-old female with mutations in the *DARS2* gene (IVS2-20_-21delTTinsC, c.374G > A) and a diagnosis of LBSL had diffuse abnormalities of the T2 signal of the cervical and thoracic spinal cord involving the dorsal columns and the lateral corticospinal tracts (patient 5).

### 3.3. Brain Imaging Findings

Brain MRI imaging that was performed on the five patients with spinal cord involvement revealed the following abnormalities: a generalized cortical atrophy in the ANS patient; multiple T2-hyperintense white-matter periventricular lesions and an enhancing lesion with an ovoid/round shape in the subject affected by LHON; brain radiological findings of the LBSL patient were characterized by bilateral periventricular T2 white-matter hyperintensities, sparing the U-fibers, associated with signal abnormalities in the superior and inferior cerebellum peduncles and in cerebellar white matter with subcortical preponderance; diffuse cerebral and cerebellar white matter involvement in combination with left putamen and right globus pallidus lesions, cerebellar cortical atrophy and brainstem abnormalities were found in LS. In the m.3243A > G carrier, no pathological findings were detected.

### 3.4. Clinical and Laboratory Findings

Patient 1 (m.3243A > G mutation) had asymptomatic spinal cord lesions. On the contrary, a mild spasticity was present in the lower limbs of patient 4 (LHON), and a severe spasticity was present in the upper and lower limbs of patient 2 (LS) and 5 (LBSL). In the last two cases we also observed the Babinski sign, which is an increased deep tendon reflex and, in patient 5, absent vibratory sensation in both legs below the knees. Finally, with regard to the ANS patient with a *POLG* mutation, we can hypothesize that the spinal-cord atrophy contributes to the ataxic phenotype. A cerebrospinal fluid analysis was performed on three patients: patient 4 revealed the presence of oligoclonal bands, while it was normal in patient 1 and 2. There were no significant common features (e.g., diet, previous treatments) among the patients with spinal-cord involvement.

## 4. Discussion

In patients with proven mitochondrial diseases, the abnormalities of the CNS are well known for cerebral and cerebellar involvement with frequently reported MRI findings [3]. Spinal cord abnormalities are rarely described in these neurogenetic disorders, with the exception of LBSL, which is a rare autosomal recessive mitochondrial disease that is associated with mutations in the *DARS2* gene and characterized by a slowly progressive pyramidal and cerebellar dysfunction, presenting a distinct and uniform MRI pattern with signal abnormalities in the cerebral white matter and the selective involvement of specific brainstem and spinal-cord tracts (dorsal columns and lateral corticospinal tracts) [10]. Furthermore, case studies have described patients affected by LHON and multiple sclerosis (MS). This association is defined as “LHON-MS”, which is a distinct clinical phenotype with specific differences from LHON and MS, but also in this case the spinal cord abnormalities are rarely reported [5,11]. Similarly, spinal cord involvement rarely characterizes subjects with MELAS or Leigh syndrome [12,13,14]. The few publications in which this clinical aspect has been examined in detail were small case studies or retrospective studies of pediatric or juvenile patients. In particular, three different research articles recently reported the high prevalence of spinal cord involvement in a case series of Kearns-Sayre syndrome (54.5%, 6/11) and cohorts of pediatric patients (74%, 14/19 and 58%, 19/33), respectively [7,8,9]. Our cross-sectional study analyzed the prevalence and the radiological characterization of spinal cord involvement in adult patients with genetically defined mitochondrial diseases. The prevalence of neuroradiological abnormalities was 9.8% and the five patients were characterized by different clinical phenotypes and mutations (MIDD, LS, ANS, LHON and LBSL were associated with m.3243A > G, m.13513G > A, *POLG*, m.11778G > A and *DARS2* mutations, respectively; Table 2). Although the prevalence in our cohort was lower than documented in pediatric or juvenile populations, it confirmed that spinal cord lesions are common in primary mitochondrial diseases. The different prevalence of spinal cord involvement between the pediatric and adult population reflects the well-known data of frequent CNS involvement in the first group that is often characterized by clinically more severe phenotypes and with a worse prognosis. Furthermore, our data confirmed the coexistence of different pathogenic mechanisms that underlie this clinical aspect, reporting both a case in which the autoimmunity played a key role (LHON-MS patient), as also suggested by the presence of oligoclonal bands in the cerebrospinal fluid, and a case in which the degenerative mechanisms play a predominant role (ANS). Interestingly, spinal-cord atrophy without signal alterations was described in a subject affected by mitochondrial diseases (patient 3). This spinal MRI feature has been previously reported in other genetic disorders such as hereditary spastic paraplegia, Friedreich’s ataxia or adult polyglucosan body disease, but never in patients with a primary defect in mitochondrial oxidative phosphorylation [6]. This observation is supported by postmortem findings in a *POLG* patient with gait ataxia characterized by posterior column degeneration [15]. Regarding the latter aspect, compared to the spinal cord more data are available on the biogenetics of the brain. In particular, the neuron-rich grey matter has a very high ATP turnover (30 μmol ATP/g tissue per min) [16] and the white matter consumes approximately one third of the energy of the gray matter [17]. Therefore, it is clear that the CNS is metabolically very demanding and consequently vulnerable to defects of the mitochondrial respiratory chain [2].

## 5. Conclusions

Our cross-sectional study analyzed the prevalence of the spinal cord involvement in a cohort of adult patients with primary mitochondrial disorders. The analysis of the data suggests that this clinical aspect is not infrequent and that the combination of brain and spinal MRI imaging associated with other symptoms suggestive of these neurogenic disorders is useful for a correct molecular diagnosis, which remains a considerable diagnostic challenge even in the omics era.

## Figures and Tables

**Table 1:** **Clinical, demographic and molecular features in patients with mitochondrial diseases undergoing spinal-cord MRI**.

Phenotype	Number of Patients	Sex	Age (y Range)	Genotype
PEO	21	7 M, 14 F	18–78	sDel, mDel, *POLG*, *TWNK*, *SLC25A4*, m.3243A > G
ANS	3	1M, 2 F	50–61	*POLG*
MELAS	3	2 M, 1 F	32–62	m.3243A > G
MIDD	6	2 M, 4 F	40–52	m.3243A > G
MERRF	7	1 M, 6 F	18–58	m.8344A > G, m.8356T > G, m.8356T > C
LHON	3	1 M, 2 F	19–45	14484T > C, m.11778G > A
ADOA	2	1 M, 1 F	42–45	*OPA1*
LS	1	F	18	m.13513G > A
LBSL	1	F	41	*DARS2*
MNGIE	2	2 M	24–44	*TYMP*
Others	2	1 M, 1 F	18–50	m.3243A > G, m.13042G > A

PEO, progressive external ophthalmoplegia; ANS, ataxia neuropathy spectrum; MELAS, mitochondrial encephalomyopathy, lactic acidosis and stroke-like episodes; MIDD, maternally inherited diabetes and deafness; MERRF, myoclonic epilepsy with ragged-red fibers; LHON, Leber hereditary optic neuropathy; ADOA, autosomal dominant optic atrophy; LS, Leigh syndrome; LBSL, leukoencephalopathy with brainstem and spinal cord involvement and lactate elevation; MNGIE, mitochondrial neurogastrointestinal encephalomyopathy.

**Table 2:** **Clinical diagnoses, genetic and magnetic resonance imaging findings in patients with mitochondrial disorders and spinal-cord involvement**.

Patient	Sex	Age at Onset	Age at MRI	Mutations	Phenotype	Spinal Cord Abnormalities
1	M	28	45 years	m.3243A > G	MIDD	cystic-like lesion located in the white matter of dorsal and lateral columns
2	F	11	18 years	m.13513G > A	LS	hyperintensity not involving a selective spinal tract with medullary atrophy
3	F	50	63 years	*POLG*	ANS	medullary atrophy without signal alterations
4	F	27	27 years	m.11778G > A	LHON	selective WMH of dorsalcolumns and lateral columns
5	F	8	41 years	*DARS2*	LBSL	hyperintensity of dorsalcolumns

MIDD, maternally inherited diabetes and deafness; LS, Leigh syndrome; ANS, ataxia neuropathy spectrum; LHON, Leber hereditary optic neuropathy; LBSL, leukoencephalopathy with brainstem and spinal cord involvement and lactate elevation; WMH, white matter hyperintensity.

## Data Availability

The data presented in this study are available on request from the corresponding author.
